# Safety and efficacy of Y-90 microsphere treatment in patients with primary and metastatic liver cancer: The tumor selectivity of the treatment as a function of tumor to liver flow ratio

**DOI:** 10.1186/1479-5876-5-15

**Published:** 2007-03-14

**Authors:** Seza A Gulec, Geraldine Mesoloras, William A Dezarn, Patrick McNeillie, Andrew S Kennedy

**Affiliations:** 1 Goshen Cancer Institute, Goshen, IN, USA; 2 Wake Radiology Oncology, PLLC, Cary, NC, USA

## Abstract

**Background:**

Treatment records and follow-up data on 40 patients with primary and metastatic liver malignancies who underwent a single whole-liver treatment with Y-90 resin microspheres (SIR-Spheres^® ^Sirtex Medical, Lake Forest, IL) were retrospectively reviewed. The objective of the study was to evaluate the anatomic and physiologic determinants of radiation dose distribution, and the dose response of tumor and liver toxicity in patients with liver malignancies who underwent hepatic arterial Y-90 resin microsphere treatment.

**Methods:**

Liver and tumor volume calculations were performed on pre-treatment CT scans. Fractional tumor and liver flow characteristics and lung shunt fractions were determined using hepatic arterial Tc-99m MAA imaging. Absorbed dose calculations were performed using the MIRD equations. Liver toxicity was assessed clinically and by liver function tests. Tumor response to therapy was assessed by CT and/or tumor markers.

**Results:**

Of the 40 patients, 5 had hepatocellular cancer (HCC), and 35 had metastatic liver tumors (15 colorectal cancer, 10 neuroendocrine tumors, 4 breast cancer, 2 lung cancer, 1 ovarian cancer, 1 endometrial cancer, and 2 unknown primary adenocarcinoma). All patients were treated in a salvage setting with a 3 to 80 week follow-up (mean: 19 weeks). Tumor volumes ranged from 15.0 to 984.2 cc (mean: 294.9 cc) and tumor to normal liver uptake ratios ranged from 2.8 to 15.4 (mean: 5.4). Average administered activity was 1.2 GBq (0.4 to 2.4 GBq). Liver absorbed doses ranged from 0.7 to 99.5 Gy (mean: 17.2 Gy). Tumor absorbed doses ranged from 40.1 to 494.8 Gy (mean: 121.5 Gy). None of the patients had clinical venoocclusive disease or therapy-induced liver failure. Seven patients (17.5 %) had transient and 7 patients (17.5 %) had persistent LFT abnormalities. There were 27 (67.5%) responders (complete response, partial response, and stable disease). Tumor response correlated with higher tumor flow ratio as measured by Tc-99m MAA imaging.

**Conclusion:**

Doses up to 99.5 Gy to uninvolved liver are tolerated with no clinical   venoocclusive disease or liver failure.   The lowest tumor dose producing a detectable response is 40.1 Gy. The utilization of MAA-based imaging techniques to determine tumor and liver blood flow for clinical treatment planning and the calculation of administered activity may improve clinical outcomes.

## Background

Yttrium-90 (Y-90) microsphere Selective Internal Radiation Treatment (SIRT), via hepatic arterial administration, is emerging as a promising treatment modality in the management of patients with primary and metastatic liver cancer [1-6]. Selectivity of the procedure is due to the unique pattern of hepatic arterial flow providing the overwhelming majority of the tumor blood supply.

The technique was introduced by Ariel [7], who also reported the first series of successful treatment in patients with metastatic colorectal cancer (CRC) [8,9]. Ariel's patients were treated with intra-arterial chemotherapy and 100 to 150 mCi (3.7 to 5.5 GBq) of Y-90 resin microspheres. The estimated radiation dose to the liver from 100 mCi (3.7GBq) of Y-90 microspheres was 120 to 180 Gy using the Medical Internal Radiation Dosimetry (MIRD) approach. This treatment modality has been included in the nuclear medicine literature since its early applications [10-12]. It has taken a few decades since the early experience in the nineteen sixties and seventies to refine the manufacturing technology and administration techniques of Y-90 microspheres before more structured studies have been implemented [13-16].

Initial treatment indications were CRC metastases and hepatocellular carcinoma (HCC), mostly for palliation, however successful application of microspheres to a variety of solid tumors is expanding the accepted indications to include other unresectable metastatic liver tumors [17-19]. More importantly, down-sizing/down-staging of hepatic tumors as a bridge to subsequent surgical treatment appears promising. The development of highly sophisticated techniques of administration has improved the therapeutic efficacy while minimizing the adverse effects [20]. Compared to the growing clinical experience with Y-90 microsphere therapy, anatomic and biologic factors affecting treatment outcomes are still not clearly described. In this study we have retrospectively reviewed the clinical data on patients who underwent whole-liver SIRT with Y-90 resin microspheres and investigated the pivotal function of tumor perfusion as quantified by Tc-99m macroaggregated albumin (MAA) imaging.

## Methods

### Radiopharmaceutical

All patients were treated with Y-90 Resin microspheres, SIR-Spheres^® ^(Sirtex Medical Inc, Lake Forest, IL). Resin microspheres have a relatively consistent diameter within the range of 35 ± 5 μm. The microspheres are biocompatible but not biodegradable, remaining permanently in the terminal arterioles of tumor vasculature and at the portal triad vessels in the normal liver following administration into the hepatic artery. Yttrium-90 (Y-90) has a half-life of 2.67 days and decays to stable Zirconium-90. It is a pure beta emitter with an average energy of 0.9337 MeV, and a mean tissue penetration of 2.5 mm (maximum range: 11 mm). Y-90 is permanently embedded within the resin structure. No significant amount of Y-90 leaches in the patient from the resin microspheres. A standard dose of resin microspheres is 2 GBq, containing approximately 50 million microspheres (range of 40 to 80 million).

### Clinical Protocol

Patients with unresectable primary and metastatic liver disease comprise the study population. All patients had unresectable disease with histologic confirmation of the primary cancer.

Patients were evaluated for adequate performance status (ECOG Performance Status score of less than or equal to 2), bone marrow reserve (granulocytes >1500/μl, platelets >60,000/μl), and hepatic function (total bilirubin <2.0 mg/dl, ALT/AST, or Alkaline   Phostphatase less than 5 times the upper limit of normal). The patients underwent a four-phase liver scan (non-contrast, arterial, portal, and equilibrium phases) for the assessment of liver disease and the evaluation of extrahepatic metastatic disease. MRI and FDG-PET imaging were performed as clinically indicated. Disease-specific tumor markers (CEA for CRC patients, AFP for HCC patients, Serotonin, chromogranin-A, 5HIAA levels for Carcinoid patients) were obtained in all patients.

Patients with acceptable liver and renal function underwent a visceral angiogram to evaluate vascular anatomy. Branches of the hepatic artery to the GI tract, such as gastroduodenal artery and right gastric artery, were sometimes coil-embolized if they posed a threat to reflux down to the stomach or duodenum At the completion of the angiogram, the patients were injected with 4 mCi (0.15 GBq) of Tc-99m MAA via the hepatic artery catheter for planar and SPECT localization imaging. Tc-99m MAA images were used to determine the lung shunt fraction (LSF) and to evaluate the extrahepatic GI localization. Patients with a lung shunting of greater than 20% and any uncorrectable GI uptake by Tc-99m MAA imaging were not treated.

The activity to be administered for each patient was determined by the empiric (Equation 1) or body surface area (BSA) method (Equation 2).

Empiric method

Tumor volume < 25% total liver volume = 2 GBq

Tumor volume 25–50% total liver volume = 2.5 GBq     Equation 1

Tumor volume >50% total liver volume = 3 GBq

Body surface area method

Dose (GBq)=BSA−0.2+VolumetumorVolumetumor+Volumeliver     Equation 2
 MathType@MTEF@5@5@+=feaafiart1ev1aaatCvAUfKttLearuWrP9MDH5MBPbIqV92AaeXatLxBI9gBaebbnrfifHhDYfgasaacH8akY=wiFfYdH8Gipec8Eeeu0xXdbba9frFj0=OqFfea0dXdd9vqai=hGuQ8kuc9pgc9s8qqaq=dirpe0xb9q8qiLsFr0=vr0=vr0dc8meaabaqaciaacaGaaeqabaqabeGadaaakeaacqqGebarcqqGVbWBcqqGZbWCcqqGLbqzcqqGGaaicqGGOaakcqqGhbWrcqqGcbGqcqqGXbqCcqGGPaqkcqGH9aqpcqqGcbGqcqqGtbWucqqGbbqqcqGHsislcqaIWaamcqGGUaGlcqaIYaGmcqGHRaWkdaWcaaqaaiabbAfawjabb+gaVjabbYgaSjabbwha1jabb2gaTjabbwgaLnaaBaaaleaacqqG0baDcqqG1bqDcqqGTbqBcqqGVbWBcqqGYbGCaeqaaaGcbaGaeeOvayLaee4Ba8MaeeiBaWMaeeyDauNaeeyBa0Maeeyzau2aaSbaaSqaaiabbsha0jabbwha1jabb2gaTjabb+gaVjabbkhaYbqabaGccqGHRaWkcqqGwbGvcqqGVbWBcqqGSbaBcqqG1bqDcqqGTbqBcqqGLbqzdaWgaaWcbaGaeeiBaWMaeeyAaKMaeeODayNaeeyzauMaeeOCaihabeaaaaGccaWLjaGaaCzcaiabbweafjabbghaXjabbwha1jabbggaHjabbsha0jabbMgaPjabb+gaVjabb6gaUjabbccaGiabikdaYaaa@7D66@

The patients underwent a whole-liver treatment following the standard administration technique. Follow-up laboratory tests included liver function tests (alkaline Phosphatase, ALT, AST, and total bilirubin) and tumor markers. The patients were followed at two and four weeks post treatment, and then monthly for three months. The National Cancer Institute's Common Terminology Criteria for Adverse Events (CTCAE v.3) was used in the grading of the toxicities, and the Response Evaluation Criteria In Solid Tumors (RECIST) criteria was used for the assessment of tumor response to treatment on CT images. The final evaluation was performed three months post treatment. PET scan response criteria were based on visual non-quantitative interpretation.

### Dosimetry

**Figure 1:**
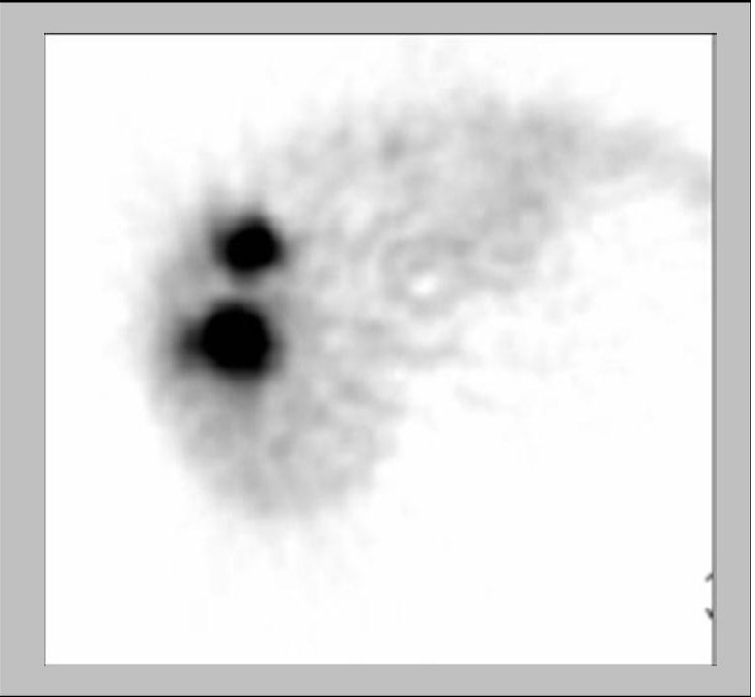
Single-Photon Emission Computed Tomography (SPECT) image from a Tc-99m MAA scan used to calculate the TLR.

The Medical Internal Radiation Dosimetry (MIRD) schema was used in the calculation of the absorbed dose in the tumor, un-involved liver, and lung compartments. The volume determinations were performed on CT scans using Pinnacle3 treatment planning software (Philips Medical), and the activity distribution within individual compartments was quantified using Tc-99m MAA Planar and SPECT images. The formulas used to calculate the LSF and the tumor to normal liver ratio (TLR) are given as Equations 3 and 4 respectively. LSF calculations are performed using the Planar image set and TLR calculations are prepared on the SPECT image set (Figure 1).

Lung Shunt Fraction

LSF=Lung countsLung counts+liver counts     Equation 3
 MathType@MTEF@5@5@+=feaafiart1ev1aaatCvAUfKttLearuWrP9MDH5MBPbIqV92AaeXatLxBI9gBaebbnrfifHhDYfgasaacH8akY=wiFfYdH8Gipec8Eeeu0xXdbba9frFj0=OqFfea0dXdd9vqai=hGuQ8kuc9pgc9s8qqaq=dirpe0xb9q8qiLsFr0=vr0=vr0dc8meaabaqaciaacaGaaeqabaqabeGadaaakeaacqqGmbatcqqGtbWucqqGgbGrcqGH9aqpdaWcaaqaaiabbYeamjabbwha1jabb6gaUjabbEgaNjabbccaGiabbogaJjabb+gaVjabbwha1jabb6gaUjabbsha0jabbohaZbqaaiabbYeamjabbwha1jabb6gaUjabbEgaNjabbccaGiabbogaJjabb+gaVjabbwha1jabb6gaUjabbsha0jabbohaZjabgUcaRiabbYgaSjabbMgaPjabbAha2jabbwgaLjabbkhaYjabbccaGiabbogaJjabb+gaVjabbwha1jabb6gaUjabbsha0jabbohaZbaacaWLjaGaaCzcaiabbweafjabbghaXjabbwha1jabbggaHjabbsha0jabbMgaPjabb+gaVjabb6gaUjabbccaGiabiodaZaaa@6CB8@

Tumor to liver ratio

TLR=Max tumor counts/pixelAverage liver counts/pixel     Equation 4
 MathType@MTEF@5@5@+=feaafiart1ev1aaatCvAUfKttLearuWrP9MDH5MBPbIqV92AaeXatLxBI9gBaebbnrfifHhDYfgasaacH8akY=wiFfYdH8Gipec8Eeeu0xXdbba9frFj0=OqFfea0dXdd9vqai=hGuQ8kuc9pgc9s8qqaq=dirpe0xb9q8qiLsFr0=vr0=vr0dc8meaabaqaciaacaGaaeqabaqabeGadaaakeaacqqGubavcqqGmbatcqqGsbGucqGH9aqpdaWcaaqaaiabb2eanjabbggaHjabbIha4jabbccaGiabbsha0jabbwha1jabb2gaTjabb+gaVjabbkhaYjabbccaGiabbogaJjabb+gaVjabbwha1jabb6gaUjabbsha0jabbohaZjabc+caViabbchaWjabbMgaPjabbIha4jabbwgaLjabbYgaSbqaaiabbgeabjabbAha2jabbwgaLjabbkhaYjabbggaHjabbEgaNjabbwgaLjabbccaGiabbYgaSjabbMgaPjabbAha2jabbwgaLjabbkhaYjabbccaGiabbogaJjabb+gaVjabbwha1jabb6gaUjabbsha0jabbohaZjabc+caViabbchaWjabbMgaPjabbIha4jabbwgaLjabbYgaSbaacaWLjaGaaCzcaiabbweafjabbghaXjabbwha1jabbggaHjabbsha0jabbMgaPjabb+gaVjabb6gaUjabbccaGiabisda0aaa@7D7A@

The primary formula used to determine the absorbed dose in the liver, tumors, and lungs is given as Equation 5. The tumor and liver masses were equated to volume (tissue density = 1 g/cc), and the lung masses were related with volume by multiplying by the assumed lung density of 0.3 g/cc. The fractional uptake is defined as the fraction of the administered activity expected to be deposited within the compartment studied (tumors. normal liver tissue, or the lungs). The lung shunt fraction was used as the fractional uptake for the lungs. The MIRD equations used in this study were described in detail elsewhere [21].

Dose (Gy)=Activityadmin (GBq)×49,800×fractional uptakeMass (g)     Equation 5
 MathType@MTEF@5@5@+=feaafiart1ev1aaatCvAUfKttLearuWrP9MDH5MBPbIqV92AaeXatLxBI9gBaebbnrfifHhDYfgasaacH8akY=wiFfYdH8Gipec8Eeeu0xXdbba9frFj0=OqFfea0dXdd9vqai=hGuQ8kuc9pgc9s8qqaq=dirpe0xb9q8qiLsFr0=vr0=vr0dc8meaabaqaciaacaGaaeqabaqabeGadaaakeaacqqGebarcqqGVbWBcqqGZbWCcqqGLbqzcqqGGaaicqGGOaakcqqGhbWrcqqG5bqEcqGGPaqkcqGH9aqpdaWcaaqaaiabbgeabjabbogaJjabbsha0jabbMgaPjabbAha2jabbMgaPjabbsha0jabbMha5naaBaaaleaacqqGHbqycqqGKbazcqqGTbqBcqqGPbqAcqqGUbGBaeqaaOGaeeiiaaIaeiikaGIaee4raCKaeeOqaiKaeeyCaeNaeiykaKIaey41aqRaeGinaqJaeGyoaKJaeiilaWIaeGioaGJaeGimaaJaeGimaaJaey41aqRaeeOzayMaeeOCaiNaeeyyaeMaee4yamMaeeiDaqNaeeyAaKMaee4Ba8MaeeOBa4MaeeyyaeMaeeiBaWMaeeiiaaIaeeyDauNaeeiCaaNaeeiDaqNaeeyyaeMaee4AaSMaeeyzaugabaGaeeyta0KaeeyyaeMaee4CamNaee4CamNaeeiiaaIaeiikaGIaee4zaCMaeiykaKcaaiaaxMaacaWLjaGaeeyrauKaeeyCaeNaeeyDauNaeeyyaeMaeeiDaqNaeeyAaKMaee4Ba8MaeeOBa4MaeeiiaaIaeGynaudaaa@8702@

### Statistical Methods

Statistical analysis was performed to determine the correlation between the parameters studied using SAS Version 9 (Cary, NC). Fisher's Exact Test was used to test association between two categorical variables, ANOVA was used to test association between a categorical variable and a continuous variable, and linear regression was used to test association between two continuous variables.

## Results

### Patients and disease characteristics

Forty patients (23 men, 17 women, ages between 31 and 81) received whole liver selective internal radiation treatment. Diagnoses were CRC in 15, HCC in 5, and neuroendocrine tumors (NET) in 10 patients. Ten patients had metastatic liver disease from various other malignancies including lung and breast cancers. All patients were treated in a salvage setting with no further medical or surgical treatment options. Table 1 details the patient and disease characteristics.

**Table 1:** Patient and disease characteristics

Age	
31–80 (Mean: 59)	
Sex	
17 female 23 male	
Disease	n
Colorectal cancer	15
Hepatocellular carcinoma	5
Neuroendocrine tumors	10
Breast cancer	4
Lung cancer	2
Endometrial cancer	1
Ovarian cancer	1
Unknown primary	2

The mean tumor volume was 294.9 ± 284.4 cc (range: 15.0 to 984.2 cc). The mean liver volume was 1662.7 ± 639.0 cc (range: 898.7 to 3982.0 cc). The mean percent tumor involvement was 14% ± 12% (range: 1% to 48%). The mean TLR by Tc-99m MAA was 6.1 ± 2.8 (range: 2.8 to 15.4). The mean lung shunt fraction was 3.3% ± 2.8% (range 0.8% to 14%). There was no difference between the anatomic and functional findings among different disease types (Table 2).

**Table 2:** Anatomic and functional findings according to disease type

Numbers in parentheses specify the range				
Anatomic and functional findings	Disease type			

	HCC	CRC	NET	All

	374.6	175.05	460.1	294.93
Tumor volume (cc)	(33.2–857.8)	(15.7–719.9)	(15.0–984.2)	(15.0–984.2)
	1592.5	2250.3	1678.5	1662.7
Liver volume (cc)	(1125.9–3982.0)	(898.7–2194.0)	(906.0–3331.8)	(898.7–3982.0)
	13.2%	8.8%	20.8%	14%
Tumor involvement (%)	(2.9–30.9%)	(0.9–33.6%)	(1.1–48.5%)	(0.9–48.5%)
	7.0	6.8	5.9	6.1
Tumor to normal liver ratio	(3.9–9.2)	(2.9–15.4)	(3.5–11.1)	(2.8–15.4)
	2.1%	4.3%	2.9%	3.3%
Lung shunt fraction	(0.9–3.3%)	(1.1–13.6%)	(0.8–8.8%)	(0.8–13.6%)

TLR and LSF values did not correlate with tumor volume, liver volume, and percent tumor involvement. The TLR and LSF values were not significantly different amongst the disease types (Table 3).

**Table 3:** TLR and LSF correlations with anatomic findings

	TLR	LSF
Tumor volume	p = 0.27	p = 0.22
Liver volume	p = 0.37	p = 0.22
Tumor involvement (%)	p = 0.55	p = 0.38
Disease type	p = 0.24	p = 0.32

### Tumor and liver absorbed dose correlates

**Figure 2:**
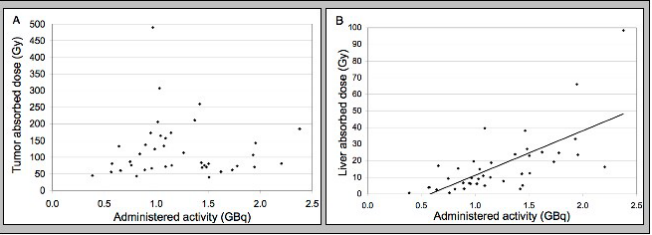
Correlation between the tumor and liver absorbed doses and the administered activity. There is a linear relationship between the administered activity and the liver absorbed dose with an equation of y = 26.80x - 15.55 and an R-squared value of 0.46. No such relationship is seen between the administered activity and the tumor absorbed dose.

**Figure 3:**
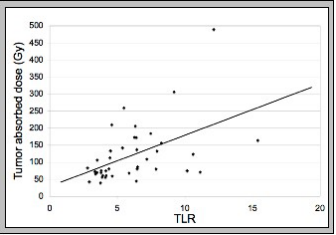
There is a linear relationship between TLR and tumor absorbed dose with an equation of y = 15.02x.+29.06 and an R-squared of 0.24. The TLR is determined using Tc-99m MAA SPECT imaging.

Administered activities for the Y-90 resin microspheres range from 0.4 to 2.4 GBq (mean: 1.2 ± 0.5 GBq). The mean absorbed doses for the tumor, liver, and lungs were 121.5 ± 85.6, 17.2 ± 18.6, and 2.1 ± 2.3 Gy respectively. No linear relationship was found between the administered activity and tumor absorbed dose. However, the liver absorbed dose increased with administered activity. Figure 2 demonstrates the administered activity-absorbed dose relationships for tumor (A) and liver (B). Table 4 summarizes the correlations between tumor and liver dose with anatomic/functional findings and administered activity.  The TLR had a linear relationship with the tumor absorbed dose with an equation   of y= 15.02x + 29.06 and an R-squared of 0.24. Figure 3 demonstrates the TLR-  absorbed dose relationship.  

**Table 4:** Tumor and liver dose correlations with anatomic/functional findings and administered activity

	Tumor dose (Gy)	Liver dose (Gy)
Tumor involvement (%)	p = 0.003*	p < 0.0001*
Tumor to liver ratio	p < 0.0001*	p = 0.47
Administered activity	p > 0.99	p < 0.0001*
Disease type	p = 0.65	p = 0.20

### Tumor and liver response correlates

The absorbed doses delivered to the tumors ranged from 40.1 Gy to 494.8 Gy (mean: 121.5 ± 85.6 Gy). Partial response or disease stabilization was observed in 27 (67.5%) patients. Median tumor absorbed doses for responders and non-responders were 107.8 Gy and 76.9 Gy respectively. The lowest tumor absorbed dose producing a detectable response was 40 Gy.  The tumor response rates for patients with CRC, HCC, and NET were 47%, 80%, and 100% respectively. Tumor response by dose is shown in Table 5 and tumor response by disease is detailed in Table 6. 

**Table 5:** Relationship between tumor absorbed dose and tumor response

Tumor response	Patients	Tumor dose (Gy)	
		
		Average	Median
Responders	27	120.3	107.8
Non-responders	13	123.9	76.9

**Table 6:** Tumor response by disease type

Disease	Patients	Tumor absorbed dose (Gy)	Tumor response
Colorectal cancer	15	136.7 (43.4–494.8)	47%
Hepatocellular carcinoma	5	135.2 (57.0–310.0)	80%
Neuroendocrine tumors	10	123.6 (40.1–262.7)	100%
Other	10	89.6 (56.2–208.4)	60%

Liver absorbed doses ranged from 0.7 Gy to 99.5 Gy (mean: 17.2 ± 18.6 Gy). Seven patients (17.5 %) had transient, and 7 patients (17.5 %) had persistent liver function test (LFT) abnormalities. The appearance of LFT abnormalities did not correlate with diagnosis, liver volume, or liver dose. None of the patients had clinical radiation hepatitis or therapy-induced liver failure. The liver response/toxicity data is shown in Table 7.

**Table 7:** Relationship between liver absorbed dose and liver response

		Liver dose (Gy)	
Liver response	Patients	Average	Median

No significant LFT change	25	12.4	9.4
Transient LFT abnormalities	7	37.0	23.9
Persistent LFT abnormalities	8	15.2	13.7

## Discussion

Radiation-associated hepatic injury has been the major hindrance in the treatment of liver malignancies using external beam radiotherapy. The incidence of potentially lethal radiation hepatitis is approximately 75% with doses in excess of 40 Gy to the whole liver using conventional external beam radiation treatment (RT) [22]. Therefore the tumoricidal dose is difficult to achieve using external beam RT.  Conformal and stereotactic radiation therapy techniques can be used to deliver much higher radiation doses for treatment of focal disease[23]; however, since hepatic metastases are most often multifocal and irregular in shape and may replace large parts of the liver volume, only a minority of patients are optimal candidates for such therapies.

The therapeutic ratio with SIRT, compared to external beam RT, is significantly improved, and the tumor absorbed doses from SIRT are typically 4 to 6 times higher than those to the liver tissue [24]. The tumoricidal dose with SIRT has not been clearly determined, and the liver absorbed doses, clinically judged to be tolerated well, are mostly based on coarse dosimetric estimates. Although the clinical efficacy of SIRT in producing objective responses, with a relatively wide margin of safety, has been clearly demonstrated in numerous studies [4,5,13-16,25], a safe liver absorbed dose has not been defined. The liver absorbed doses reported in the literature range from 34 to 181 Gy [25-29]. This variability is primarily due to the differences in the dosimetric methodologies adopted. It is further accentuated with the heterogeneity in the available retrospective data that involves different disease types with different underlying functional liver abnormalities and previous treatments.

Microspheres are deposited in the liver as a number of discrete clusters, rather than point sources that are homogeneously distributed throughout the tumor. This irregular but non-random clustering of microspheres produces a highly heterogeneous radiation dose distribution pattern. Microsphere distribution in and around tumors is complex. Microspheres lodge preferentially in the growing rim of the tumor. The central portion of lesions, often times hypoperfused and sometimes necrotic, receive a much smaller number of microspheres, if any. The variability in the dose distribution with Y-90 microsphere treatment has been emphasized in a number of studies [24,30,31]. The uncertainty of dose deposition is very unsettling for both conventional brachytherapy, and the MIRD methodology of nuclear medicine.

Microspheres distribute within tumor and liver compartments. The differential deposition of microspheres favoring the tumor compartment is expressed as the TLR. The "selectivity" of the Y-90 microsphere treatment is determined primarily by the TLR, the biologic determinants of which include the angiogenic potential and pattern of the tumors, and the growth rate-dependent tumor perfusion. The TLR, in our study, did not correlate with tumor volume, liver volume, or percent tumor involvement. There was no correlation between the TLR and tumor type within the observed range of 2.8 to 15.4. The TLR had a linear relationship with the tumor absorbed dose with an equation of y = 15.02x + 29.06 and an R-squared of 0.24.

Currently, MAA scans are performed routinely prior to SIRT for the assessment of lung shunting and extrahepatic uptake. This is widely recognized to be important for minimizing the risk of potentially debilitating adverse events such as radiation pneumonitis or radiation gastritis/duodenitis. MAA particles are of similar size and density as resin microspheres (MAA   particles have diameters in the range 15 to 30 microns and a density of 1.3   g/cc and SIR-Sphere microspheres have diameters of 35 ± 5 microns and a density of 1.6 g/cc). Although the number of particles for MAA and resin microsphere suspensions (in a unit of administered volume) is significantly different (microspheres are concentrated 10-fold) and the potential effect of this difference on the distribution kinetics has never been studied, it is reasonable to conclude that the two particles will be distributed similarly within the liver and the tumor compartments. Several studies have demonstrated the value of the MAA scan in planning of a successful SIRT [32-34]. The role of the MAA scan was challenged by Dhabuwala et al. in a recent paper which aimed to determine whether the pattern or degree of MAA uptake by liver tumors after hepatic arterial injection might be a predictor of response to subsequent SIRT with resin microspheres [35]. The authors were not able to demonstrate a correlation between MAA uptake in the CRC liver metastases prior to SIRT and response to the treatment determined by CEA, CT, or survival time. However, this study suffered serious methodological problems. The MAA was injected 1 to 2 hours prior to the SIRT. The SIRT was administered to the patients after injecting angiotensin 2 into the hepatic artery, whereas the MAA perfusion scans were performed without angiotensin 2. The effects of the incomplete clearance of MAA on subsequent Y-90 microsphere injection and the application of angiotensin 2 pharmacological manipulation to the therapy session but not to the MAA administration confounded the results. The use of simple planar images for the quantitative element of the study brought a significant degree of imprecision to the measurement of the MAA uptake as well.

The theory of SIRT is based on a favorable TLR. MAA imaging is a good measure of TLR, despite its significant technical limitations. A high resolution SPECT acquisition and processing is essential in MAA image interpretation and quantitation. The avascular core of the lesions needs to be excluded from the activity calculation during quantitative image processing for improved accuracy. The MAA directly shows the site and the amount of anticipated radiation dose deposition. The vascularity of different tumor types has been studied extensively. A vascularity grade as assessed by an arteriogram may not necessarily predict the TLR measured from an MAA scan. The wide range of TLR for many types of tumors also indicate that, based on the histologic type, the targeting efficiency of Y-90 microspheres (or MAA) may not be correctly estimated. The individual TLR is determined by the stage and the pattern of angiogenesis, and also by synchronous events of apoptosis and necrosis. There are no scientific grounds for precluding any particular type of liver malignancy for SIRT, nor is there a credible way of predicting favorable tumor targeting without MAA imaging. The correlation between the TLR and clinical outcome may be more difficult to establish as the clinical response is determined by many other factors. The radiosensitivity of the tumor (intrinsic or microenviromental), therapeutic threshold, and the tumor growth rate-to-necrosis/apoptosis ratio could play significant role in producing objective tumor responses.

The second important observation in our study was related to the relationships between tumor and liver absorbed doses and administered activity. The tumor absorbed dose did not correlate with the administered activity. However, there was a linear correlation between the liver absorbed dose and the administered activity.

The estimation of the tumor absorbed dose with Y-90 microspheres using MIRD macrodosimetry approach is problematic. The non-uniformity of microsphere distribution is not only a function of irregular tumor angio-architecture, but also is complicated with flow-bound distribution physics. Although the total dose delivered to the tumor compartment can be determined reasonably accurate, the dose deposition pattern in the different zones within a given tumor mass does challenge the limits of macrodosimetry. The angiogenic belt at the tumor-normal tissue boundary has the highest and relatively uniform microsphere distribution. A full radiation dose deposition occurs in this zone while an inner zone receives an attenuated dose from the far range beta emission of the former. The radiation deposition within deeper tumor regions is drastically lower than that at the periphery.

The inhomogeneity in the distribution of the microspheres in the liver tissue is to a lesser scale when compared to the distribution of microspheres in the tumor. Radiation dose to healthy liver parenchyma is determined by the number of microspheres administered, the specific activity of the microspheres, and the distance between the microspheres implanted. The liver tissue receiving the highest dose is that which is immediately surrounding the tumor. The radiation injury to this area of parenchyma is more pronounced. There is an abrupt drop in the radiation dose towards the surrounding liver a few millimeters from the high dose zone around the tumor [31]. The remainder of the liver receives less radiation than would be predicted from assuming a homogeneous distribution of radiation dose throughout the parenchyma. Fox et al. have demonstrated that almost 90% of the liver tissue received less than the dose predicted by assuming uniform distribution, and a third of the tissue received less than one-third of the predicted dose [24]. This partially explains the lack of classical clinical radiation hepatitis at much higher dose levels as estimated by MIRD methodology. The linear relationship between administered activity and liver dose observed in our study indicates that MIRD estimates could be clinically useful in defining safe liver dose limits. The application of microdosimetry techniques will improve the reproducibility of the radiation dose calculations. This clearly requires higher resolution functional diagnostic imaging.

The third set of observations in our study was related to the dose response in tumor and liver. Objective tumor response was seen in more than 50% of all patients. There was no difference in mean absorbed tumor dose estimates amongst the disease types. The differences in response could perhaps be explained by variations in radiosensitivity. This result could also be partly due to the differences in the techniques used to assess the treatment response. FDG-PET response usually is observed before a volume change is appreciated on CT imaging [36]. Similarly, tumor markers and/or octreoscan changes might be more sensitive indices of objective response for NET compared to CT imaging alone. A concrete conclusion regarding the differences in objective response amongst disease categories may not be drawn with a restricted sample size.

There was no classical clinical radiation hepatitis or therapy-induced liver failure at liver absorbed doses up to 99 Gy. In 63% of the patients, there were no significant LFT changes and in 18%, the initial LFT abnormalities were improved in 2 to 4 weeks. A grade 1 to 2 rise in LFT values was not uncommon following treatments associated with angiographic evidence of stasis at the end of the microsphere administration.

The liver response (toxicity) to radiation appears to be different in SIRT than in external beam RT. This is mainly due to the lower dose rates maintained by virtue of the selectivity of Y-90 microsphere treatment. The differences in the severity and pattern of liver toxicity could be related to the radio-patho-biologic differences in both techniques. The pathogenesis of radiation damage from external beam RT to the liver is dominated by vascular injury in the centri-lobular region. External beam radiation causes alterations in the central veins such as eccentric wall thickening and subsequent obliteration of the lumen leading to veno-occlusive disease [37]. Radiation from microspheres, however, is deposited primarily in the portal triads. The relative segregation of the central vein from the radiation source was suggested to be important in minimizing the toxicity due to venoocclusive disease [26]. However, the toxicity related to the radiation effects on the biliary tract and distal portal tributaries should be approached with due caution.

## Conclusion

An analysis of radiation distribution in the tumor and liver compartments indicated that the tumor absorbed dose best correlated with tumor-to-liver Tc-99m MAA uptake ratio supporting the clinical value of quantification of activity distribution and dose calculations before the administration of Y-90 microsphere treatment. The majority of patients exhibited an objective treatment response. Liver absorbed doses up to 100 Gy were well tolerated with no clinical venoocclusive disease or liver failure. The lowest tumor dose producing a detectable response was 40 Gy. The utilization of MAA-based imaging techniques to determine tumor and liver blood flow for clinical treatment planning and the determination of administered activity may improve clinical outcomes. The accuracy of dose estimates will improve parallel to the improvements in the techniques of image quantitation for determination of activity distribution.

## Authors' contributions

SG participated in the conception and design of the study, analysis and interpretation of data, drafting the manuscript, and the critical revision of the manuscript. GM participated in the analysis and interpretation of data and the drafting of the manuscript. AD participated in the acquisition of the data, the interpretation of the data, and the critical revision of the manuscript. PM participated in the acquisition of the data. AK participated in the conception and design of the study, analysis and interpretation of data, and the critical revision of the manuscript.
